# Differential Correlation of Transcriptome Data Reveals Gene Pairs and Pathways Involved in Treatment of *Citrobacter rodentium* Infection with Bioactive Punicalagin

**DOI:** 10.3390/molecules28217369

**Published:** 2023-10-31

**Authors:** Damarius S. Fleming, Fang Liu, Robert W. Li

**Affiliations:** 1 USDA-ARS, Beltsville Agricultural Research Center, Animal Parasitic Diseases Laboratory, Beltsville, MD 20705, USA; robert.li@usda.gov; 2 Zhengzhou University, Zhengzhou 450001, China; fliu19910205@gmail.com

**Keywords:** parasite, citrobacter, bioactive compound, differential correlation

## Abstract

This study is part of the work investigating bioactive fruit enzymes as sustainable alternatives to parasite anthelmintics that can help reverse the trend of lost efficacy. The study looked to define biological and molecular interactions that demonstrate the ability of the pomegranate extract punicalagin against intracellular parasites. The study compared transcriptomic reads of two distinct conditions. Condition A was treated with punicalagin (PA) and challenged with *Citrobacter rodentium*, while condition B (CM) consisted of a group that was challenged and given mock treatment of PBS. To understand the effect of punicalagin on transcriptomic changes between conditions, a differential correlation analysis was conducted. The analysis examined the regulatory connections of genes expressed between different treatment conditions by statistically querying the relationship between correlated gene pairs and modules in differing conditions. The results indicated that punicalagin treatment had strong positive correlations with the over-enriched gene ontology (GO) terms related to oxidoreductase activity and lipid metabolism. However, the GO terms for immune and cytokine responses were strongly correlated with no punicalagin treatment. The results matched previous studies that showed punicalagin to have potent antioxidant and antiparasitic effects when used to treat parasitic infections in mice and livestock. Overall, the results indicated that punicalagin enhanced the effect of tissue-resident genes.

## 1. Introduction

Resistance to drug treatments for infectious microbes has been a growing trend across many disease-causing organisms. From bacterial to viral infections, research has been undertaken to address the waning efficacy of antibiotics and vaccines [1,2]. The same issue is present in the world of parasites, where traditional anthelmintics used to protect livestock have begun to also lose efficacy over time [1,2]. The issue of treatment resistance swells in complexity when multiple pathogens of concern become unaffected by broad-spectrum treatments that once worked across disease categories. As agriculturists worldwide strive to keep their eyes open to new and emerging threats and treatments to livestock parasite pathology, there is also a need improve protection for current livestock parasites that are advancing quicker than traditional anthelmintics. To this end, the study and use of bioactive fruit extracts (BFEs) as alternatives to anthelmintics has shown promise as a stand-alone treatment and complement to traditional anthelmintics. Chemical extracts of multiple organic acids and terpenes have shown antimicrobial efficacy against bacterial infections of the gut. Additionally, extracted phenolic compounds in the form of tannic acids and ellagitannins have exhibited not only antimicrobial actions [3] but also antiparasitic ability [4,5]. One of these fruits with extracts that showed promise is the pomegranate (*Punica granatum*). The pomegranate plant and the products of its fruit have been studied for decades for their health benefits as a food source for humans. It has also been studied for the benefit of its extracts for their immunomodulation, antiparasitic, antioxidant, and apoptotic impacts on animal health. Pomegranate peel extracts have been confirmed to be effective against parasites like Giardia and Eimeria in mouse trials [6,7] and performed as well as commercial anthelmintics in cattle, sheep, goat, and buffalo. To examine the antiparasitic ability of other BFEs present in pomegranate, our study takes a look at the polyphenol punicalagin [8,9]. Studies have shown that punicalagin exhibits potent antioxidant and anti-inflammatory capabilities against bacterial and parasitic infections [9,10,11]. The effectiveness of punicalagin may be attributed to its tannin content, which is thought to produce potent anthelmintic-like actions against intestinal nematodes [12]. Smith et al., 2020, examined the use of pomegranate peel extracts against Citrobacter infections and Stefanou et al., 2021, further expanded on the use of pomegranate and its antibacterial actions against a bevy of pathogens [13,14]. Unlike previous studies, our study examines only the purified punicalagin’s impact on the host reaction to the bacteria *Citrobacter rodentium* at the genomic level. By studying the differences in gene expression and differential gene correlation, we were able to uncover gene interactions that show that punicalagin effectively protects the host from parasite infection and collateral damage to the host from its immune response.

## 2. Results

### 2.1. Differential Expression of PA vs. CM

The results from the differential expression analysis showed a total of 1448 (734 upregulated/714 downregulated) expressed genes in the PA vs. CM that met the significance threshold of a q-value ≤ 0.05. The normalized counts for all individuals were used to interpret biological interactions based on the treatment groups. All downstream results reported in this manuscript are based on a gene matrix of the individuals across each condition, PA (condition A) or CM (condition B). Of the top five upregulated genes, three were pseudogenes and non-informative. The two informative genes in the top five were Mettl7a2, now known as thiol methyltransferase 1A2 (Tmt1a2; Log2fc = 6.54), located in lipid droplets and predicted to be involved in DNA/mRNA methylation and methyltransferase activity. The other gene was cytochrome P450, family 2, subfamily c, polypeptide 69 (Cyp2c69; log2fc = 3.25), that enables the catalysis and conversion of acid into various compounds active in the epoxygenase P450 pathway thought to produce viable anti-inflammatory and vasodilatory impacts in humans. The gene also facilitates oxidoreductase and caffeine oxidation functions [15,16,17]. The top five downregulated genes consisted of only one pseudo gene. The four annotated genes included the unc-5 family C-terminal-like (Unc5cl; log2fc = −23.49), a positive pro-inflammatory signal regulator, and Cyp3a44 cytochrome P450, family 3, subfamily a, polypeptide 44 (Cyp3a44; log2fc = −16.08), involved in lipid hydroxylation and oxidative demethylation activity. The last two genes, small proline-rich protein 2G (Sprr2g; log2fc = −3.34), part of the creation of the host epidermal barrier, and lipid binding and chitinase-like 3 (Chil3; log2fc = −2.71), functionally similar to bacterial chitinases, were less downregulated [15,16,17]. 

### 2.2. Top Differentially Correlated Genes with Condition A (PA) across Classes +/0 and +/− 

The genes were ranked according to the number of class-specific gene pair correlations for a single gene vs. all others. The analysis was restricted to reporting only the top 200 genes in each classification. The two gene classifications, +/0 and +/−, are based on the correlations observed for each treatment group. The first symbol of the classifications refers to the treatment group administered the punicalagin (PA), and the second symbol refers to the group not fed the punicalagin (CM). For the +/0 classification, the “+” symbol refers to the gene with a positive correlation with the PA group, while “0” refers to no correlation in the compared CM group. The classification of +/− also refers to the PA and CM groups, respectively. The “+” here again refers to a positive correlation with PA, and the “−” refers to the gene wit a negative correlation with the CM group. For the class +/0, the top genes with the highest number of positively correlated interactions for condition A (PA) were hemoglobin, beta adult t chain (Hbb-bt; N = 658), hemoglobin alpha, adult chain 1 (Hba-a1; N = 638), hemoglobin, beta adult s chain (Hbb-bs; N = 627), and hemoglobin alpha, adult chain 2 (Hba-a2; N = 619). Annotations for the function and processes of these four genes is related to oxygen binding and carrier activity, peroxidase activity, and bacterium responses [15,16,17]. Other genes of interest in the +/0 class have functions that bridge antioxidants, metabolism, nutrient delivery, and immunity. This included the genes folate receptor beta (Folr2; N = 593), T cell lymphoma invasion and metastasis 1 (Tiam1; N = 522), retinol-binding protein 7, cellular (Rpb7; N = 551), lysyl oxidase-like 3 (Loxl3; N = 482), and vanin 1(Vnn1; N = 456) [15,16,17]. The next class examined was +/−, showing gene pairs that were positively correlated to condition A vs. being negatively correlated with condition B. The gene at the top of this list was perilipin 1 (Plin1; N = 91). Genes in the +/− classification had overall lower numbers of formed gene pairs. Because the software allows genes to overlap different classes, many genes, like Hbb-bs, Folr2, and Siglec1, appeared in both classifications (Table 1).

### 2.3. Top Differentially Correlated Gene Pairs from Classes +/0 and +/−

Genes were selected from the top differentially correlated gene pairs across classes +/0 and +/− for analysis of the unique gene vs. all others and presented the key gene pair correlations for the top DC genes. Differential correlation analysis returned results in the top 200 most significant gene pairs with a focus on gene pairs positively correlated with condition A. Individual genes were then selected from that list and analyzed to determine the differential correlation between that gene and all other genes in the matrix. Of the 200 gene pairs, 44 were classified as positively correlated with condition A (PA) and had no correlation or a negative one with condition B (CM) (Table 2).

### 2.4. Module-Based Differential Correlation of Genes between Condition A and Condition B

Results emphasized which genes from the 64 detected modules had the strongest gains and losses in connectivity between the punicalagin extract fed mice and the untreated mice given both were infected with *C. rodentium*. The modules that met the permuted *p*-value threshold (0.8) (Table 3) showed gain of correlation for multiple genes related to antioxidant, antimicrobial, and possible host immune strengthening processes of bioactive enzymes of pomegranate. Modules with positive correlation with condition A (PA) (Table 4) were plotted against those with an overall negative correlation to condition A (positive correlated to condition B) based on measure of the modular average differential connectivity (MeDC) values. 

### 2.5. Top GO Terms Differentially Correlated with Modules with Positive Correlation with PA Treatment

**Figure 1:**
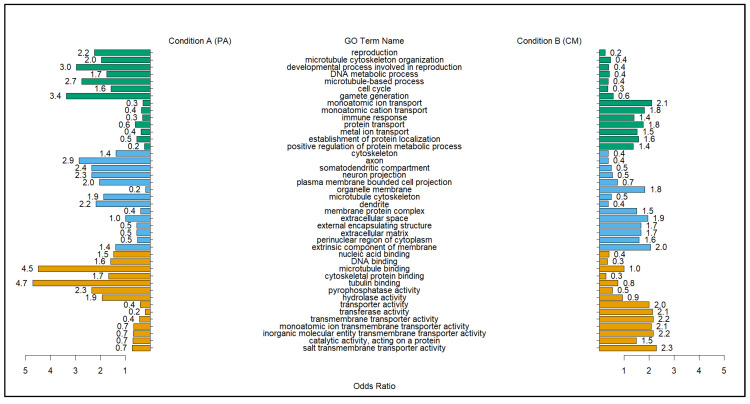
GO term comparison between the highest positively and negatively correlated modules. Highlighted terms are linked to some of the reported effects of pomegranate extracts like punicalagin on parasitic infection. Modules c1_80 (positive correlation, MeDC = 1.07) and c1_202 (negative correlation, MeDC = −1.65). Green = biological process (BP), blue = cellular compartment (CC), and yellow = molecular function (MF) represent categories for the GO terms displayed.

**Figure 2:**
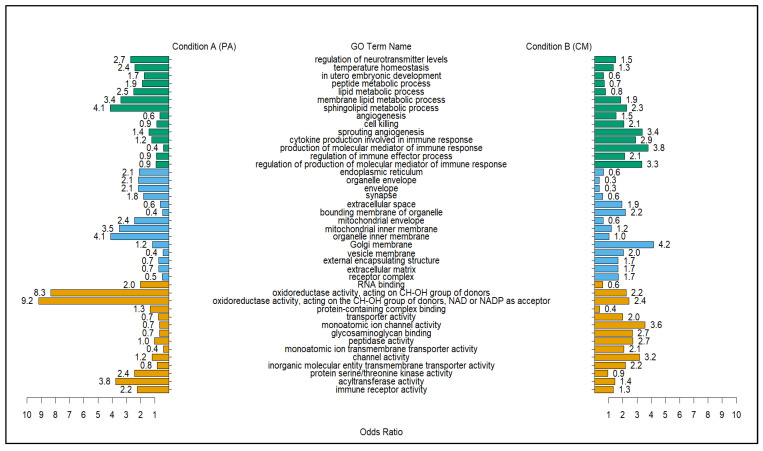
Comparison of module c1_123 and c1_202. Modules c1_123 (positive correlation, MeDC = 0.70) and c1_202 (negative correlation, MeDC = −1.65). Comparison of the module c1_123, which contained multiple heme genes that were shown to be positively correlated with punicalagin treatment. Green = biological process (BP), blue = cellular compartment (CC), and yellow = molecular function (MF) represent categories for the GO terms displayed.

Data derived for gene ontology (GO) differential correlation were produced using the –ddcorGO, –ModuleGO functions in the DGCA package. The function uses the overall gene pair correlations within a given module to identify terms. The modules that had an overall median differential connectivity (MeDC) showing that the majority of genes within a given model were positive correlates of punicalagin treatment were examined for their enriched GO terms. Results from the gene-module-based correlation test of the 64 modules yielded only 18 that were positively correlated and met the liberal permutation threshold of 0.8 (*n* = 100). Plotting the results of the differentially correlated gene modules was used to visualize the connectivity of GO terms diverging in correlation between conditions. The use of the MeDC values provided a metric for comparing modules that were positively correlated with treatment with punicalagin against Citrobacter and those that were negatively correlated. The five modules that displayed the strongest positive correlation were c1_80, c1_104, c1_207, c1_123, and c1_39; the five that showed the least correlation were c1_202, c1_49, c1_27, c1_24, and c1_212. Overall, the degree of connectivity was stronger for the negatively correlated modules (Table 3). The modules with the strongest gain and loss of connectivity, c1_80 and c1_202, were plotted against each other to see what GO terms linked to the modules differed by treatment (Figure 1). This plotting was also performed between c1_202 (negative DC) and c1_123, which contained genes that were shown to be highly correlated in gene pairs, as well as the modules (Figure 2). 

A comparison of modules c1_80 and c1_202 showed that the positively correlated GO terms for condition A (PA) center around reproductive and general growth processes. The terms included reproduction (GO:0000003), cell cycle (GO:0007049), DNA binding (GO:0003677), and pyrophosphatase activity (GO:0016462). However, the GO terms that were more correlated with condition B (CM) indicated that the untreated and infected mice (CM) experienced a higher biological burden. This was represented by the term immune response (GO:0006955), multiple protein and transmembrane transporter activity terms, and the cellular compartment terms of external encapsulating structure (GO:0030312). The latter were connected to bacterial infections in mice [18,19]. Module comparison between c1_123 and c1_202 expanded on the immune-related differences between conditions. All of the immune action terms were strongly correlated with condition B, denoting the issues that the CM treatment group had with battling the parasite. Correlated with no punicalagin treatment were the immune function terms cell killing (GO:0001906) and cytokine production involved in immune response (GO:0002367), with one of strongest correlation differences being for the term production of molecular mediator of immune response (GO:0002440). In contrast with condition B (CM) mice, treatment with punicalagin had a strong correlation with terms related to temperature homeostasis (GO:0001659), lipid metabolic process (GO:0006629), and multiple redox reactions that included oxidoreductase activity, acting on the CH-OH group of donors, NAD or NADP as acceptors (GO:0016616), and acyltransferase activity (GO:0016746) (Figure 2).

## 3. Discussion

Bioactive fruit enzymes may provide sustainable alternatives to parasite anthelmintics. Here, we sought to define biological and molecular interactions that are enhanced by administering a pomegranate extract (punicalagin) to mice infected with *Citrobacter rodentium*. Differential correlation analysis revealed coordinated changes in the expression of gene pairs and modules. Punicalagin treatment induced strong positive correlations with genes related to oxidoreductase activity and lipid metabolism. Conversely, immune and cytokine responses characterized untreated, infected mice. These responses to bacterial infection in mice evidently model effects induced by punicalagin in livestock infected with parasites, suggesting opportunities for pursuing this extract as a tool to control a range of infectious agents.

### 3.1. Annotated Functions of Positively Correlated Top Genes and Gene Pairs Indicated That Punicalagin-Treated Mice Had a Broad Spectrum-like Impact against C. rodentium

When the genes that make up the list of genes that are positively differentially correlated with condition A (PA) were examined individually, different genes, with similar functions to the differential expression analysis, were strongly correlated. The DEG showed that the highest up- and downregulated genes functioned within the host in mRNA methylation, methyltransferase, oxidoreductase, lipid, and anti-inflammatory activity. The positively correlated gene pairs were also annotated within mice as performing these activities when infected mice were treated with dietary supplements of punicalagin extracts. The genes that fell into the category of being positively correlated with the treatment but not the untreated group (+/0) included a series of hemoglobin genes that play roles in the transfer and binding of oxygen as well as peroxidase activity. The genes themselves (Hbb-bt, Hbb-bs, Hba-a1, and Hba-a2) are located in cell lipid droplets, which are thought to be both a source for host metabolic responses to intracellular parasites and critical to parasites that rely on host lipids for cholesterol [20,21]. When other top differentiated genes in the +/0 class are examined along with the series of heme activity genes, we start to see a picture emerging of what processes punicalagin extract appears to benefit. Some of the genes that showed up in the list of forming the most correlations, such as Rbp7, Siglec1, and Folr2, also appeared in the list of top gene pairs positively correlated with treatment with punicalagin. The genes formed pairs with tenascin XB (Txnb), Zbtb20, and sulfotransferase family 1A, phenol-preferring, and member 1 (Sult1a1). The paired Rpb7 is shown to be an integral part of the endothelial response to oxidative stress [22], which complements the antioxidant attributes of pomegranate bioactive enzymes against other parasites like Giardia and Eimeria [6,7]. The gene paired with Rbp7, Txnb, is a collagen- and fibronectin-binding cell-adhesion molecule that is active in the extracellular matrix and in mice is ubiquitously expressed throughout the gastrointestinal tract [15,17]. It is possible that this gene pair is coordinated through their interaction with peroxisome-proliferator-activated receptor gamma (Pparℽ) as part of host metabolic or anti-inflammatory functioning. The gene pair of Siglec1 and Zbtb20 could be related to immune system activity. The gene Siglec1 is mostly known as a viral receptor but also T cell and apoptotic functions; while Zbtb20 plays roles in lipid homeostasis, negative regulation of transcription, and the regulation of multiple cytokines. The link between the genes may be due to interaction in the intestine with T cells [23]. The last gene–gene pair of Folr2 and Sult1a1 have functions that indicate that punicalagin treatment impacts metabolic features correlated with nutrient uptake within the gastrointestinal system [15,17]. Differential correlation of multiple gene pairs appeared to show that punicalagin enhanced the effect of tissue-resident genes against *C. rodentium*. From a gene pair perspective, many of the actions attributed to genes mirror actions of current drug treatments by signaling apoptotic activity, interfering with lipid synthesis, redox reactions, and other inhibitory measures [2,12].

### 3.2. PA vs. CM Differentially Correlated GO Terms Based on Positively Correlated Gene Modules Further Elucidate the Effects of Punicalagin (PA) against Bacterial Parasites Compared to the Infected Untreated Group (CM)

The pathways that had significant gains of correlation within the group administered the punicalagin (PA) appeared to show protective immune responses to the *C. rodentium* infection. Two of the condition A (PA) positive correlated modules, c1_123 and c1_39, contained gains for Hbb-bs and Hbb-bt. The modules were positively correlated with condition A, but with the same single gene that lost correlation, unlike the top two strongest correlated modules c1_80 and c1_104, which were completely positively correlated. A comparison of c1_80 (+/0) and c1_202 (0/+) showed that many of the GO terms that were correlated with the pomegranate extract centered around reproduction and cell maintenance (Figure 1). This result is most likely the result of less stress to perform immune actions in this group than the result of punicalagin enhancing reproductive functions. This is reinforced by the GO term for immune response (GO:0006955) with a stronger correlation with condition B. This outcome appeared to show that punicalagin treatment allowed infected mice to maintain fertility, which could signify that less stress is being experienced at the molecular level due to punicalagin’s effects [24]. The inversely correlated terms of immune response and reproduction may further underscore the antioxidant ability of punicalagin, whose function may be involved in proper sperm and oocyte maintenance [5,6,8]. 

The biological correlations that were seen in the mice that received punicalagin in their diet by comparing c1_123 and c1_202, (Figure 2) echoed purported beneficial health effects of pomegranate in fruit, juice, and extract form [5,11,25]. The comparison of these two modules displayed multiple GO terms, indicating that punicalagin fostered homeostasis around the immune response to parasite infections. One possibility is that punicalagin increases oxygen binding through genes like Hbb-bs, Hbb-bt, Hba-a1, and Hba-a2 (Module c1_104), leading to increased peroxidase and oxidoreductase activity. The heme genes also play a role in the host bacterial response. Its correlation with treatment could be due to a boost in scavenger responses driven by the antioxidant properties of punicalagins phenolic compounds [8,10,26]. There is some indication of anti-inflammatory effects with positive correlation with the GO term temperature homeostasis (GO:0001659), showing that punicalagin-treated mice had better control over their core temperatures, lessening the chance of cell-destructive fevers. The group treated with the extract also experienced an increase in metabolic processes related to lipids and peptides (lipid metabolic process, GO:0006629), (sphingolipid metabolic process, GO:0006665), (peptide metabolic process, GO:0006518). The link may be with fatty acid utilization via the host or parasite. However, the skewing of the immune function GO terms toward the untreated group (Figure 2) indicates that the metabolic activity is improved by the inclusion of punicalagin. This could be an indication of host uptake of the antioxidant and antiparasitic metabolites in punicalagin [4,10,11]. The strongest-correlated molecular function term was centered on oxidoreductase activity (GO:0016616), further indicating that punicalagin was linked to improved antioxidant functions. Although the modulation of the dosage may be needed to fully inhibit bacterial parasites, the dosage used within this study showed evidence of a lessened molecular cost for the host. In livestock, this could serve as a model for examining parasite resilience over resistance. Terms that were positively and negatively correlated with the punicalagin-treated mice mirrored previous transcriptome studies that showed that gene co-expression networks that were related to pro-inflammatory cytokine responses were also downregulated, while metabolism and neuroactive receptor activity were upregulated [11]. The number, strength of correlation, and enriched GO terms of the gene pairs and modules provided new insight into the antiparasitic capabilities of plant bioactives. Differential correlation becomes a measure of how well or poorly the untreated and infected group is faring. In this study, it gave context to those molecular interactions that are favorably associated with previously reported effects of pomegranate extract on parasite infections. 

## 4. Materials and Methods

### 4.1. Animals

A total of 30 mice were randomized into three treatment groups (*n* = 10) based on the compound used to protect against a Citrobacter rodentium-induced colitis model. The treatment groups consisted of an uninfected, PBS-supplemented control group labeled “NC”. The other treatment groups were both infected with the bacterium and supplemented with the PBS and labeled as group “CM”, or with the experimental natural bioactive punicalagin and labeled as “PA”. Groups NC and CM were used as negative and positive controls against PA, respectively. The mice strain used in this experiment was C3H/HeNCr. Mice groups were fed a basal diet with the tested dietary supplements (punicalagin, PBS) administered for a total of 15 days, which consisted of 3 days prior to infection and 12 days post-infection (dpi) for all 3 treatment groups. The purified punicalagn (water-soluble) and PBS were given as dietary supplements as an oral gavage of 200 uL. Colons were then collected from the mice after 12 dpi for RNA extraction. 

We used two preparations of punicalagin in the related studies. One is pure punicalagin purchased from Biosynth International, Inc., San Diego, CA, USA (Cat# FP74904 with a purity of >98%). Another formulation was purified in-house from pomegranates (*Punica granatum*) using a patented procedure described in CN101974043B. The purity was evaluated using HPLC at approximately 97.5%. Both alpha and beta anomers coexist in the final product. Both preparations were similar in efficacy. 

The experiment was conducted under IACUC approval of animal protocol# 18-027 and procedures were implemented according to the animal-use protocols approved by the USDA Beltsville Area Institutional Animal Care Committee. The *C. rodentium* production and administration to the mice was performed using the method reported in Liu et al., 2023 [11]. The results of comparing the PA group (infected and given punicalagin to treat infection) and the CM group (infected and given PBS in place of punicalagin) are presented in this manuscript. The comparison between the PA and CM groups allowed us to observe the changes in immune action that the addition or lack of punicalagin had on bacterial infections. For the purposes of this study, the groups are also referred to as condition A (PA) and condition B (CM).

### 4.2. RNA Sequencing and Expression Analysis

Colon tissue samples were extracted from the colons of the mice using the Illumina TruSeq total RNA kit™. Paired-end (PE) 100 bp sequences were produced using an Illumina Novaseq 6000™. The PE sequences were put through quality control, mapped, and analyzed for differential expression using the Hisat2-StringTie-DeSeq2 pipeline default parameters using the method reported in Liu et al., 2023 [11,27,28,29,30]. Gene annotations were based on Ensembl database records for mouse strain C3H/HeNCr [15]. Final gene lists were based on a significance threshold set at q-value ≤ 0.05. The normalized read counts from these data were used downstream for differential correlation (DC) analysis.

### 4.3. Differential Correlation and Gene Ontology Analysis

To better understand the different effects of the DEGs within and between treatment groups, a differential correlation analysis was conducted using the R package DGCA [31]. The analysis allowed for the examination of the regulatory connections of genes expressed between different treatment conditions by statistically querying the relationship between correlated gene pairs related to differing conditions. The correlation metric used within DGCA was the default Pearson’s correlation since the RNA-seq size factors were similar across all samples. Differential correlation was used to find biological interactions related to the transcriptomic changes across the study conditions, treated with punicalagin or without treatment, during *Citrobacter rodentium* infections. Differential correlation between the 3 treatment conditions was conducted with the R packages DGCA and MEGENA [31,32]. Input for DGCA consisted of pre-processed normalized gene counts for all samples (*N* = 30) that resulted from the differential expression analysis method reported in Liu et al., 2023 [11]. The matrix of normalized read counts for each sample was used as the base input for DGCA. Formatting for DGCA input was further modified by translating ensemble gene IDs into the gene names and removing all duplicate gene names and NAs (*N* = 3651) and all genes whose rows totaled less than 100 normalized reads (*n* = 28,908). This left a total of 22,279 genes for input. Another filter was completed within DGCA using the package matrixStats and the argument FilterGenes to filter the list for centrality to the median (filterCentralPercentile = 0.35) and low-value dispersion measurements based on the coefficient of variation (filterDispersionPercentile = 0.7). Filtering using the central tendency and dispersion resulted in a final list of 4341 genes. The threshold for correlation was set to 0.99, and the *p*-value for significant gene pair correlation was set at 0.8 (default = 1) permutations (*N* = 10) using –ddcorAll. The function –getCors was then used to find the correlation values within each condition based on the default Pearson correlation to build a correlation matrix object. The matrix object was then used as input for the –pairwiseDCor function to calculate the differential correlation between conditions and calculate its significance. To examine the most significant differentially correlated gene pairs between the conditions, the treatment groups for this study are referred to as “conditions”. Expression results were tested to identify differential correlation between gene pairs as well as the correlation of single genes vs. sets of multiple genes between conditions. A list of the top differentially correlated genes based on Z scores was compiled using the –topDCGenes function. Genes in the list were ranked according to the total number of gene pairs formed for each of the correlation classes (*N* = 9), Z score difference (up and down), and “all”. Next, the top correlated gene pairs based on the pairwise comparison were captured using the –dcTopPairs function for the top 200 correlated pairs. Tables were constructed to classify the gene pair correlation results into groups based on the differential connectivity of the genes to the conditions examined. Gene pairs were classified into 1 of 9 groups, with the results presented in this study focused only on gene pairs that had a positive correlation in condition A, no significant correlation in condition B (+/0) and a positive correlation in condition A, and negative correlation in condition B (+/−). Initial GO analysis was carried out on individual genes, where the gene pair positively correlated with condition A (treated with punicalagin) using –ddcorGO function in DGCA. Individual genes were selected from a ranked list of outputs of –topDCGenes and –dcTopPairs.

### 4.4. Differential Correlation Enrichment of Gene-Specific and Module-Based Gene Ontology (GO)

In order to examine the molecular pathways and functions that were positively correlated with punicalagin treatment, both individual genes and genes clustered into modules were examined for GO over-enrichment. The DGCA integration with the R package MEGENA was used to first examine the differential correlation results (*N* = 4341 genes, n = 2 conditions) and then organize them into modules. Modules were based on the do.MEGENA command defaults for *p*-value cut-off (0.05) to classify genes as “hub” or “nodes” based on permutations. Module edges and vertices were based on planar filter network (PFN) calculations. Modules produced in MEGENA were implemented within DGCA. For the gene-specific GO analyses, an adjusted *p*-value of 0.05 was used as the significance threshold. The function –ddcorAll with the splitSet argument was used to calculate the correlation between a given gene of interest and every other gene in the list. To detect the positively correlated GO terms, the function –ddcorGO was used and the resulting terms were sorted into 1 of 9 correlation classes, with reporting of the +/0 and +/– of condition A (PA). The modules’ output from MEGENA was then used as input into the function –ModuleDC to ascertain the modules that contained genes with strong gains or losses in the correlation between condition A (PA) and condition B (CM). Modules with the overall strongest gains in connectivity were used as the input for the –moduleGO function to find the related gene ontology (GO) terms correlated with condition A (PA). The top module-based GO terms used an unadjusted *p*-value of 0.05. The modules that only had gain of-correlation (GOC) genes were used from the module-based analysis. Genes with the strongest gain or loss of connectivity for treatment with punicalagin were also examined. All plots were made within DGCA. For all analyses, the ceiling for correlation was set to 0.99.

## 5. Conclusions

Overall, this study uncovered interactions between gene pairs that were positively correlated with treatment with punicalagin. The correlations of these gene pairs offer new opportunities to find new biomarkers related to antiparasitic activity. Combining the gene pair results with those of the gene modules that were positively correlated potentially enables the discovery of biological connections that may be the result of small changes across multiple correlated gene networks. The differential correlation analysis indicated that punicalagin treatment had strong positive correlations with the over-enriched gene ontology (GO) terms related to oxidoreductase activity and lipid metabolism. Conversely, the GO terms for immune and cytokine responses were strongly correlated with no punicalagin treatment. Although our study used a bacterial pathogen, many of the positively correlated results matched previous studies that show that punicalagin has potent antioxidant and antiparasitic effects when used to treat bacterial or parasitic infections in mice and livestock.

## Figures and Tables

**Table 1:** The top 15 differentially correlated genes across classes +/0 and +/− for condition A. These are the genes that formed the most gene pairs in relation to being positively correlated to condition A (PA) treatment with the phenolic compound punicalagin and either have no or a negative correlation to condition B (CM). # of DC pairs = total number of gene-pairs formed in each class.

	Genes in Class +/0	# of DC Pairs	Genes in Class +/−	# of DC Pairs
1	Hbb-bt	658	Plin1	91
2	Hba-a1	638	Folr2	68
3	Hbb-bs	627	Gk	55
4	Hba-a2	619	Siglec1	54
5	Folr2	593	Gpr179	51
6	Rbp7	551	Hbb-bs	48
7	Tiam1	522	Fabp4	46
8	Cbfa2t3	500	1700003D09Rik	44
9	Siglec1	489	Gm20634	43
10	1700003D09Rik	488	Hbb-bt	43
11	Loxl3	482	Kctd9	43
12	Vnn1	456	H2ac18	41
13	Smad5	453	Plin4	33
14	Wdr35	449	Phtf1os	31
15	Abtb2	447	Zbtb12	31

**Table 2:** The top 30 positive correlated gene pairs between the infected, treated with bioactive punicalagin (PA), and the infected, untreated (CM) groups. There were only a total of 44 positively correlated gene pairs out the top 200 reported by DGCA. Permuted *p*-values = 0.05 (PvalAdj) were set as significance threshold. Classes (+/0) means gene pair positively correlated with condition A (PA), and no correlation in condition B (CM); (+/−) means gene pair positively correlated with condition A and negatively correlated with condition B.

Gene1	Gene2	corA (PA)	corA_pVal (PA)	corB (CM)	corB_pVal (CM)	zScoreDiff	pValDiff	pValDiff_adj	Classes
Gm16185	Exoc3l4	0.975	1.75 × 10^−6^	−0.725	1.76 × 10^−2^	−5.79	6.92 × 10^−9^	3.74 × 10^−7^	+/−
Kctd9	Smim30	0.867	1.16 × 10^−3^	−0.937	6.20 × 10^−5^	−5.68	1.33 × 10^−8^	4.42 × 10^−7^	+/−
Mgll	Gpr179	0.957	1.36 × 10^−5^	−0.799	5.52 × 10^−3^	−5.63	1.77 × 10^−8^	4.75 × 10^−7^	+/−
Styx	Foxn3	0.909	2.63 × 10^−4^	−0.902	3.65 × 10^−4^	−5.62	1.90 × 10^−8^	4.75 × 10^−7^	+/−
Ttc41	Pald1	0.982	4.69 × 10^−7^	−0.521	1.23 × 10^−1^	−5.47	4.57 × 10^−8^	7.61 × 10^−7^	+/0
Rbp7	Tnxb	0.983	3.62 × 10^−7^	−0.483	1.57 × 10^−1^	−5.44	5.46 × 10^−8^	8.41 × 10^−7^	+/0
Gm37240	Mgll	0.992	1.83 × 10^−8^	−0.239	5.07 × 10^−1^	−5.41	6.42 × 10^−8^	8.94 × 10^−7^	+/0
Boc	Gpr179	0.951	2.29 × 10^−5^	−0.741	1.43 × 10^−2^	−5.24	1.65 × 10^−7^	1.25 × 10^−6^	+/−
mt-Tt	Mgll	0.990	3.59 × 10^−8^	−0.142	6.96 × 10^−1^	−5.22	1.81 × 10^−7^	1.25 × 10^−6^	+/0
Coch	Tiam1	0.981	6.14 × 10^−7^	−0.437	2.07 × 10^−1^	−5.20	1.99 × 10^−7^	1.27 × 10^−6^	+/0
Adra2a	H2ac18	0.796	5.93 × 10^−3^	−0.929	1.01 × 10^−4^	−5.12	3.01 × 10^−7^	1.43 × 10^−6^	+/−
Timd4	Ltk	0.986	1.53 × 10^−7^	−0.243	4.99 × 10^−1^	−5.12	3.10 × 10^−7^	1.43 × 10^−6^	+/0
Kif12	Gpr179	0.965	6.19 × 10^−6^	−0.608	6.24 × 10^−2^	−5.09	3.56 × 10^−7^	1.43 × 10^−6^	+/0
Nptn	Chp1	0.994	4.68 × 10^−9^	−0.073	8.40 × 10^−1^	−5.09	3.60 × 10^−7^	1.43 × 10^−6^	+/0
Smad5	Ifi27	0.960	1.03 × 10^−5^	−0.633	4.95 × 10^−2^	−5.05	4.53 × 10^−7^	1.51 × 10^−6^	+/−
Siglec1	Adam23	0.961	9.45 × 10^−6^	−0.622	5.47 × 10^−2^	−5.03	4.82 × 10^−7^	1.53 × 10^−6^	+/0
Folr2	Gm12216	0.938	5.90 × 10^−5^	−0.745	1.34 × 10^−2^	−5.02	5.09 × 10^−7^	1.57 × 10^−6^	+/−
Airn	Prkar2b	0.897	4.28 × 10^−4^	−0.831	2.87 × 10^−3^	−4.96	7.06 × 10^−7^	1.87 × 10^−6^	+/−
Cd69	Eml5	0.993	1.08 × 10^−8^	0.001	9.99 × 10^−1^	−4.95	7.40 × 10^−7^	1.87 × 10^−6^	+/0
Gk	Tns4	0.644	4.44 × 10^−2^	−0.953	2.05 × 10^−5^	−4.91	8.95 × 10^−7^	1.91 × 10^−6^	+/−
Gpr18	Tiam1	0.974	1.98 × 10^−6^	−0.433	2.11 × 10^−1^	−4.91	8.98 × 10^−7^	1.91 × 10^−6^	+/0
Gm42743	Ifi27l2a	0.865	1.22 × 10^−3^	−0.860	1.40 × 10^−3^	−4.88	1.05 × 10^−6^	2.03 × 10^−6^	+/−
Celf3	Fcna	0.969	3.84 × 10^−6^	−0.481	1.60 × 10^−1^	−4.87	1.13 × 10^−6^	2.10 × 10^−6^	+/0
Gm37033	Siglec1	0.872	9.91 × 10^−4^	−0.848	1.95 × 10^−3^	−4.85	1.25 × 10^−6^	2.14 × 10^−6^	+/−
Siglec1	Zbtb20	0.964	7.19 × 10^−6^	−0.533	1.13 × 10^−1^	−4.85	1.26 × 10^−6^	2.14 × 10^−6^	+/0
Rbp7	Slc16a7	0.984	2.76 × 10^−7^	−0.175	6.29 × 10^−1^	−4.84	1.28 × 10^−6^	2.14 × 10^−6^	+/0
Gbe1	Nras	0.781	7.69 × 10^−3^	−0.911	2.51 × 10^−4^	−4.82	1.41 × 10^−6^	2.18 × 10^−6^	+/−
Enpep	Gpr179	0.905	3.21 × 10^−4^	−0.790	6.59 × 10^−3^	−4.80	1.55 × 10^−6^	2.23 × 10^−6^	+/−
Samd5	Etfbkmt	0.871	1.02 × 10^−3^	−0.840	2.33 × 10^−3^	−4.79	1.65 × 10^−6^	2.24 × 10^−6^	+/−
Ccdc69	Tiam1	0.933	8.32 × 10^−5^	−0.706	2.24 × 10^−2^	−4.79	1.70 × 10^−6^	2.24 × 10^−6^	+/−

**Table 3:** Module-based list of genes with the top 5 strongest gain or loss of connectivity for treatment with punicalagin extract against Citrobacter rodentium infection. The significance threshold was set to 0.8. MeDC (median differential connectivity), GOC (gain of connectivity), LOC (loss of connectivity). Overall, there were more modules that were negatively correlated with punicalagin treatment. This could be a result of the untreated mice that were expressing more gene interaction in relation to the immune system working harder to battle the parasite infection.

Module	Size	MeDC	pVal	Top_GOC	Top_LOC
c1_80	50	1.07	0	Gpr179, Fcna, Gm37240, Hoxb5os, Rgs11, Znf41-ps, 9230105E05Rik, Gm43254, Ccdc125, Fcgr4	
c1_104	30	0.86	0	Hba-a1, Hba-a2, Fosb, Dstyk, Pkmyt1, 2700054A10Rik, Apod, Mcub, Vsig10	
c1_207	80	0.86	0.38	Tiam1, Basp1, Serpina3g, Memo1	Mt4
c1_123	75	0.70	0	Hbb-bs, Hbb-bt, Ifi27l2a, H2ac8, Ybx2, Pde1a, Mdfi, Smim6, Prss27, Fam161a	Zmiz1
c1_39	129	0.60	0	Hbb-bs, Hbb-bt, Ifi27l2a, H2ac8, Klra2, Ybx2, Pde1a, Mdfi, Prss27, Cercam	Zmiz1
c1_202	58	−1.65	0	Slc4a11	Igkv1-110, Igkv1-117, Slc16a12, Jchain, Gli1, Maob, Ces2d-ps, Rab11fip4, Igkv5-45, Chst1
c1_49	41	−1.51	0		Cdc42ep2, Nfatc1, Birc5, Thop1, Eno1b, Omp, Gm6311, Rps2-ps13, Gm12366, Gm5786
c1_27	41	−1.49	0		Atf3, Tacc2, Gpat3, Tmcc3, Tube1, Dusp10, Cdk6, Brca2, Schip1, Rasgrf2
c1_24	33	−1.45	0		Atg10, Dlst, Col6a5, Ric8b, Kif5c, Msi2, Acsl3, Abcb1b, Atp2b1, Heg1
c1_212	35	−1.40	0		Gm12320, Pou2f2, Igkv10-94, Cd180, Slc40a1, Gdpd1, Ano10, Zfp608, Phf21a, H2bc24

**Table 4:** Modules positively correlated with punicalagin treatment C. rodentium in mice. Based on permuted (*n* = 100) *p*-value set at 0.8 for examination purposes. MeDC (median differential connectivity), GOC (gain of connectivity). Only the positive correlation classes of +/0 and +/− are shown in the table.

Module	Size	MeDC	Pval	Top GOC in Condition A (PA)
C1_80	50	1.07	0	Gpr179, Fcna, Gm37240, Hoxb5os, Rgs11, Znf41ps, 9230105E05Rik, Gm43254, Ccdc125, Fcgr4
C1_104	30	0.86	0	Hba-a1, Hba-a2, Fosb, Dstyk, Pkmyt1, 2700054A10Rik, Apod, Mcub, Vsig10
C1_207	80	0.86	0.38	Tiam1, Basp1, Serpina3g, Memo1
C1_123	75	0.70	0	Hbb-bs, Hbb-bt, Ifi27l2a, H2ac8, Ybx2, Pde1a, Mdfi, Smim6, Prss27, Fam161a
C1_39	129	0.60	0	Hbb-bs, Hbb-bt, Ifi27l2a, H2ac8, Klra2, Ybx2, Pde1a, Mdfi, Prss27, Cercam
C1_10	56	0.57	0	Ttc41, Klhl4, Oxnad1, Per3, Plscr2, Dbp, 5830444B04Rik, Pald1, Prxl2a, Il18r1
C1_44	31	0.48	0.04	C4b, Tle2, Slc9a5, Tie1, Slc22a17, Zfp72
C1_149	159	0.41	0.54	Tiam1, Abtb2, Socs1, Memo1, Tchh
C1_45	47	0.36	0.12	Gm47798, Cdk5r1, Trafd1, Slc18a1
C1_48	40	0.34	0.08	Gm49322, Rac3, Fam177a, 2700038G22Rik, Il4ra
C1_151	48	0.32	0.08	Dclk2, Elavl1, Ptpro, Snx32, Mill2, Syt8, Six5, Vstm5, Septin3
C1_20	92	0.32	0.34	1700003D09Rik, Gm20634, H2ac18, Hba-a1, Hba-a2, Ablim2, Apod, Pkmyt1
C1_22	42	0.28	0.18	Loxl3, Tmcc2, 9830144P21Rik
C1_67	171	0.26	0.32	Siglec1, Plin1, Igkv4-74, Batf3, Ntng2, Car3, Timd4, Plin4, Il6ra, Exoc3l4
C1_9	89	0.26	0.26	Gpr179, Prkar2b, Fcna, Gm37240, Hoxb5os, Fcgr4, Znf41-ps, Rgs11, Ccdc125, mt-Tt
C1_25	106	0.24	0.26	Folr2, Cd209f, Hspa12b, Thrsp, Dcn, Gm21188, Plxna3, Snai1, Cavin2, Tbx21
C1_47	44	0.09	0.5	Meox1, Gm15675, BC034090, Ccr5, Scara5, Dcp1a, 5830408C22Rik, Gm43980
C1_43	59	0.07	0.72	Fabp4, Cd36, Gm36161, Ifit2

## Data Availability

List of data included in manuscript can be found at NCBI Sequence read Archive (SRA) beginning November 2023, SRA#PRJNA1035093.
